# The effect of oral progesterone for the treatment of abnormal uterine bleeding in women taking warfarin following prosthetic valve replacement

**DOI:** 10.12669/pjms.35.4.907

**Published:** 2019

**Authors:** Shafaq Nadeem, Shahid Abbas, Anjum Jalal

**Affiliations:** 1 Shafaq Nadeem, FCPS (Gynecology & Obstetrics), Department of Cardiac Surgery, The Clinic for Women with Cardiac Diseases, Faisalabad Institute of Cardiology, Faisalabad, Pakistan; 2 Shahid Abbas, FCPS (Cardiology), Department of Cardiology, Faisalabad Institute of Cardiology, Faisalabad, Pakistan; 3 Anjum Jalal FRCS, FCPS (Cardiac Surgery), FRCS-CTh, Department of Cardiac Surgery, Faisalabad Institute of Cardiology, Faisalabad, Pakistan

**Keywords:** Abnormal uterine bleeding, Menorrhagia, Polymenorrhea, Valve replacement, Warfarin induced menorrhagia

## Abstract

**Objectives:**

To evaluate the effect of oral progesterone for the treatment of abnormal uterine bleeding in patients taking warfarin after prosthetic valve replacement.

**Methods:**

A total of 85 women of reproductive age, who were on warfarin due to prosthetic valve replacement were enrolled in the study. After detailed evaluation, their menstrual bleeding was quantified using Pictorial Bleeding Evaluation Chart. The patients were then prescribed an oral progesterone (Norethisterone) 5mg three times daily. The first follow up was done after one-month then at 3-months and at six months. The improvement in PBAC score was recorded at each visit. Data was entered and analyzed using SPSS (version 23.0). The mean ± Standard Deviation were calculated for quantitative variables while qualitative variables were presented in frequency table. The normality of data was checked using Kolmogorov-Smirnov test. Due lack of normal distribution of data in various groups, the Wilcoxon Sign Rank test was used to test the significance before and after treatment. The p-value of <0.05 was taken as statistically significant.

**Results:**

The mean age of the patients was 30.13±7.69 years. The mean PBAC score was 162.8 ± 24.86 before initiation of treatment while at the end of the treatment it was 105.48 ± 8.38. Forty-six (54.1%) patients had continuous per vaginal bleeding, 33 (38.8%) had menorrhagia, 4 (4.7%) had inter-menstrual bleeding and 2 (2.4%) had menorrhagia along with polymenorrhea. The mean dose of warfarin taken by the patients was 5.85 ± 2.69 mg. The median parity of the patients was 2. The Wilcoxon Sign Rank test showed p-value of <0.00001 for comparison of the pre-treatment PCBA values with those of one, three and six-months after the treatment. The Friedman’s test also had a p-value of <0.00001. This confirmed that the post treatment bleeding was significantly less than pretreatment bleeding.

**Conclusion:**

The warfarin induced abnormal uterine bleeding can be controlled effectively and safely with low dose of oral progesterone.

## INTRODUCTION

The literature is scanty about menstrual problems among women on anticoagulants due to cardiac problems. The increased risk of abnormal uterine bleeding with Warfarin is widely established. However, the strategies to deal with this problem are deficient due to lack of coordination between cardiology and gynecology.

Oral progesterone is as efficacious as that of other hormonal treatment like combined oral contraceptive pills which are relatively less safe in such patients. Sub dermal implants are less effective for menstrual problems while intrauterine devices are invasive mode of treatment with added cost.^1^ Though, intra uterine devices provide a novel approach to tackle with this problem, these cannot be applied to unmarried girls and non-affording women. The safety profile, cost effectiveness and easy availability of oral progesterone can help to reduce various menstrual problems. The aim of this study was to evaluate the effect of oral progesterone for the treatment of abnormal uterine bleeding in patients taking warfarin after prosthetic valve replacement.

## METHODS

**Fig. 1:**
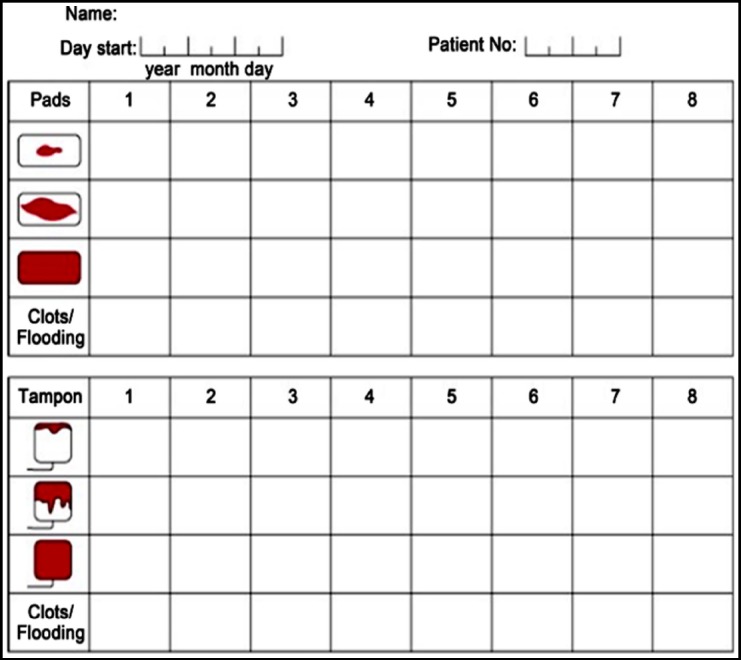
The Pictorial Bleeding Assessment Chart (PBAC).

This cross sectional descriptive study was conducted at Faisalabad Institute of Cardiology in department of cardiac surgery with the association of consultant gynecologist, cardiologist and cardiac surgeon. A total of 85 women of reproductive age, who had undergone prosthetic valve replacement were enrolled in the study. They were taking warfarin therapy for at least three months along with enteric coated aspirin 75 mg once daily. The INR was kept within the therapeutic range of 2.5 to 3.5 in all patients. At the time of recruitment in the study details of their menstrual cycle including the length, regularity, flow, and abnormal pattern bleeding were assessed using a questionnaire. Patients were counseled to evaluate the degree of saturation for each sanitary product used during menstruation. During the next menstrual period the bleeding was quantified using score sheet of PBAC (Fig.1). The total number of each type of sanitary product was then listed in the corresponding row during each day of menstruation. Scores on PBAC were assigned for each pad as shown in the Fig.1. A lightly stained pad scored one point, the moderately saturated pad was given five points and completely soaked pad was given 20 points. Moreover, presence of small clot scored one point, whereas large clot scored 5. None of patients used tampons. After noting down the bleeding in pre-progesterone menstrual period, the patients were prescribed an oral progesterone (Norethisterone) 5 mg three times daily cyclically. The first follow up was done after one-month then at 3-months and at six months. The quantification was done as described above for these follow-up visits. The patient compliance and sides effects noted by the patients were also recorded. The INR was monitored on the weekly basis and was kept within recommended therapeutic range of 2.5 to 3.5 very strictly.

The improvement in PBAC score was recorded at each visit. Data was entered and analyzed using SPSS (version 23.0). The mean ± Standard Deviation was calculated for quantitative variables like age, parity, warfarin dose and PBAC score. While qualitative variables like type of prosthetic valve and abnormal menstrual bleeding pattern were presented in frequency table. The normality of data was checked using Kolmogorov-Smirnov test. The Wilcoxon Sign Rank test was used to test the significance before and after treatment due lack of normal distribution of data in various groups detected by the Kolmogorov-Smirnov test. The p-value of <0.05 was taken as statistically significant. The Nul hypothesis was defined as: “there is no difference of PBAC score before and after the treatment with progesterone in female patients on warfarin therapy”.

## RESULTS

In this study, out of 85 women, the mean age of respondents was 30.13±7.69 years. The mean PBAC score was 162.8 ± 24.86 before initiation of treatment while at the end of the treatment it was 105.48 ± 8.38. Regarding menstrual bleeding, 46 (54.1%) had continuous per vaginal bleeding, 33 (38.8%) had menorrhagia, four (4.7%) had inter-menstrual bleeding and two (2.4%) had menorrhagia along with polymenorrhea. The cardiac surgery profile of the patients is shown in Table-I. Most of them had mitral valve replacement as expected. The mean dose of warfarin taken by the patients was 5.85 mg with standard deviation of 2.69 mg and the median dose of 5 mg. The median parity of the patients was two.

**Table I:** Frequency distribution of various variables.

Variable		Frequency	Percentage (%)
Age (Years)	< 20	10	11.8
	21-30	38	44.7
	31-40	29	34.1
	41-49	08	9.4
Menstrual bleeding	Continuous Per Vaginal	46	54.1
	Menorrhagia	33	38.8
	Inter menstrual Bleeding	04	4.7
	Menorrhagia + Polymenorrhea	02	2.4
Reason for warfarin therapy	Mitral Valve Replacement	32	37.6
	Double Valve Replacement	29	34.1
	Aortic Valve Replacement	24	28.2
	Total	85	100

Kolmogorov Smirnov test for normality of data was conducted (Table-II). This table shows the values of PBAC in two groups i.e. the pre-treatment group and 6-months following the progesterone treatment group, have p-values less than 0.05 and hence are not normally distributed. However, the values of PBAC after one and three months of progesterone treatment are normally distributed. We therefore used non-parametric test to compare the pre-treatment PBAC values with those of one, three and six-months after the treatment, individually using the Wilcoxon Sign Rank test (Table-III). It is obvious that all three comparisons showed highly significant differences as the p-values were <0.00001 in each comparison. It is also obvious from the set of data that the PBAC scores were taken from the same individuals at four different times serially. Therefore, we also tested the data with Friedman Test which is a non-parametric variant of ANOVA with repeated measures. The Friedman’s *X*^2^_r_ was 224.05 which has a p-value of <0.00001. This again proves that the downward trend of PBAC scores after progesterone treatment has a very strong statistical significance.

**Table II:** Normality Tests (Kolmogorov Smirnov Test).

	Mean PBAC	Median PBCA	Stand Deviation PBCA	K-S Statistic (D)	K-S p-value
Pre-Progesterone	162.80	162	24.86	0.190	0.0038
One month Post Progesterone	125.41	123	16.02	0.128	0.1142
3-months Post Progesterone	110.86	110	10.10	0.118	0.1700
6-months Post Progeterone	105.48	103	8.38	0.147	0.0454

**Table III:** Comparison of PBAC score before and after treatment with Progesterone using Wilcoxon Sign Rank Test.

Pre – Post Treatment	W Statistics	Mean Difference	Z Statistic	*p*
Pre vs One month Post Progesterone	0	30.8	-8.0077	<0.00001
Pre vs 3-months Post Progeterone	2.5	48.8	-7.9967	< 0.00001
Pre vs 6-months Post Progesterone	1	50.8	-8.0033	< 0.00001

The pretreatment PBAC and the final post treatment PBAC at 6-months follow-up were also compared in various subgroups of Age, Bleeding Pattern and the Surgical profile. The Wicoxon Sign Rank test for all sub group analyses showed a p-value of <0.00001 which is highly significant (Table-IV). This means the progesterone treatment was effective in all subgroups. The patients showed excellent compliance as there were no drop outs from the study. The patient did not report any untoward effects of the progesterone during the study period.

**Table IV:** Comparison of pretreatment PBAC score with those at six-months post treatment follow-up in different subgroups.

Grouping Variables	Subgroups	Mean Rank	Z Statistic	p
Age (Years)	≤ 20	5.50	-2.805	0.005*
	21-30	20.00	-5.360	< 0.001*
	31-40	15.00	-4.706	< 0.001*
	41-49	4.50	-2.512	0.012*
Menstrual Bleeding	Continuous Per Vaginal	24.00	-5.896	< 0.001*
	Menorrhagia	17.00	-5.014	< 0.001*
	Inter menstrual Bleeding	2.5	-1.826	0.068
	Menorrhagia + Polymenorrhea	1.50	-1.342	0.180
Treatment	MVR	17.00	-4.919	< 0.001*
	DVR	15.00	-4.705	< 0.001*
	AVR	12.50	-4.287	< 0.001*

* Significant Value (p-value < 0.05)

## DISCUSSION

Rheumatic heart disease (RHD) frequently takes a chronic course causing congestive heart failure, stroke, endocarditis and death.^2^ Since early 20th century the incidence of rheumatic heart disease has consistently decreased in developed countries. However, it is still a major cause of morbidity and mortality in young age in the developing countries, including Pakistan.^3^-^5^ The prevalence of RHD is more in females compared to the males, because women are house bound and therefore are more likely to be affected by overcrowding.^6^

The majority of RHD patients develop valvular heart disease. The mitral valve stenosis with or without incompetence is the most common valvular lesion seen in these patients.^7^ Patients who undergo surgical intervention may have either a bio prosthetic valve replacement or a mechanical valve replacement.^8^ After surgical treatment there is need to initiate vitamin K receptor antagonist drugs like warfarin in order to prevent thrombus formation of the mechanical valves. Since its discovery in 1940, warfarin has withstood the test of time despite many newer oral anticoagulants in the current medical scenario.^9^ Warfarin carries significant risk of bleeding and interaction with several medications.^10^ It is reported that, only 62% - 66% of the patients taking Warfarin achieve a therapeutic level.^11^ It is therefore mandatory to monitor the effect of warfarin on regular intervals. The female patients taking warfarin are likely to suffer from heavy menstrual bleeding (HMB). A study of 90 women, aged 15–49 years, reported that the abnormal uterine bleeding was reported by 17.8% women before use of anticoagulation therapy while 29.5% women reported this problem after the start of anticoagulants.^12^ The treatment of HMB is quite challenging and the efficacy of commonly used medicines like Tranexamic acid is not well established.^13^ The other pharmacological options include Levonorgestrel intrauterine system, high dose progestin-only therapy, and combined hormonal contraceptives. However, these treatments are associated with fears of thrombosis and the safety is not well established. Women who do not respond to medical treatment or who do not wish to retain their fertility may be considered for surgical management.^14^

While reporting the efficacy of any form of treatment it is necessary to use an objective method of quantification of abnormal uterine bleeding. So far there is no purely objective method to quantify the bleeding due to practical problems and hence a semi-objective approach is needed. Attempts to evolve this type of evaluation have led to development of Pictorial Bleeding Assessment Chart (PBAC). The PBAC is a tool widely used in research settings to quantify menstrual blood loss. A pictorial chart score of 100 or more has been shown to have a specificity and sensitivity of 80% when used as a diagnostic test for heavy menstrual bleeding. Huq et al. showed that 60% of women using warfarin have HMB, with a longer duration of menstruation.^15^ Warner and colleagues found positive correlations between objective menorrhagia with passing clots more than 1.1 inches in diameter and changing pads more frequently than every three hours.^16^ The PBAC score has objective value as a total score more than 100 points per menstrual cycle have been shown to indicate >80-mL of blood loss.^17^-^19^

Our study is perhaps first of its kind in Pakistan which has examined the role of oral progesterone for treatment of abnormal uterine bleeding among women on warfarin. In this study, 54.1% had continuous per vaginal bleeding, 38.8% had menorrhagia, 4.7% had inter-menstrual bleeding and 2.4% had menorrhagia along with polymenorrhea. The efficacy of norethisterone in our study has no doubts as it was found to reduce the PBAC scores across all sub groups and at all points of follow-up. It is interesting that the patients had 100% compliance during the study period. In fact, many women were extremely obliged as they never received that degree of attention from the medical community in past. However, it is premature to extrapolate that they would continue this treatment on long term basis. Similarly, no sides effects of progesterone therapy were reported in the study period. This again must be taken with caution because if this therapy is advised over the extended premenopausal period of young women, the side effect may turn out to be of significant clinical significance. The prolonged used of norethisterone is known to adversely affects the important lipoprotein risk factors for coronary heart disease as it decreases HDL cholesterol and increased LDL cholesterol levels.^20^ Similarly, it is also known to have increased tendency of venous thrombosis. Nevertheless, it is still a satisfactory option to reduce the menopausal bleeding in patients with prosthetic valves.

### Limitations of study

The study has the limitations of being non-randomized and without any placebo and blinding. Yet one can argue that all patients acted as their own controls hence it still carries a lot of significance.

## CONCLUSION

The warfarin induced abnormal uterine bleeding can be controlled effectively and safely with low dose of oral progesterone. The patients show good compliance to this mode of treatment.

### Author`s Contribution

**SK:** Conceived, designed, did statistical analysis & prepared the first draft of manuscript.

**SA:** Helped in study design and manuscript review.

**AJ:** Supervised the study, conducted analysis and did final review of manuscript.
